# Supplementary material for: A retrieval-specific mechanism of adaptive forgetting in the mammalian brain
Source: Nat Commun. 2018 Nov 7;9:4660. doi: 10.1038/s41467-018-07128-7 (PMC6220340; doi:10.1038/s41467-018-07128-7)
Supplement: Supplementary file 1 — Supplementary Information [file 41467_2018_7128_MOESM1_ESM.pdf]

Bekinschtein et al.

A Retrieval-Specific Mechanism of Adaptive Forgetting in the Mammalian Brain

Supplementary Information

Supplementary Figures 1-7
Supplementary Methods
Supplementary Tables 1-14

Supplementary Figures

A

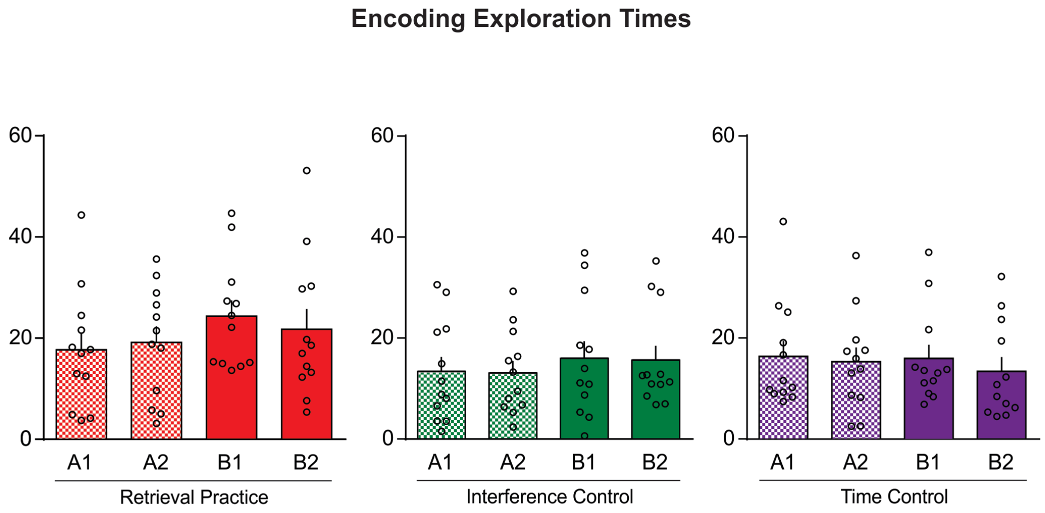

B

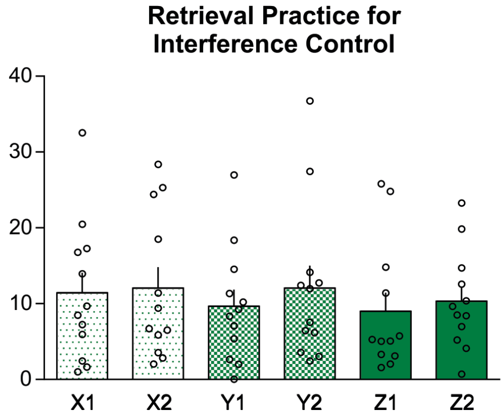

Supplementary Figure 1. Encoding and practice exploration times. (A) During encoding, the number of seconds that animals explored the to-be-practiced object A and to-be-competitor object B during the two trials (distinct letters indicate different objects; numbers indicate copies) for each of the conditions. Values are means ± SEM. There were no significant differences between exploration of one object and the paired identical object, one-tailed paired *t* test. (B) During practice, the number of seconds that animals explored the two distractor objects (on trial 1, X1 & X2; on trial 2, Y1 and Y2; on trial 3, Z1 and Z2) during the three trials for the interference control condition. There were no significant differences between exploration of one object and the paired identical object, one-tailed paired *t* test.

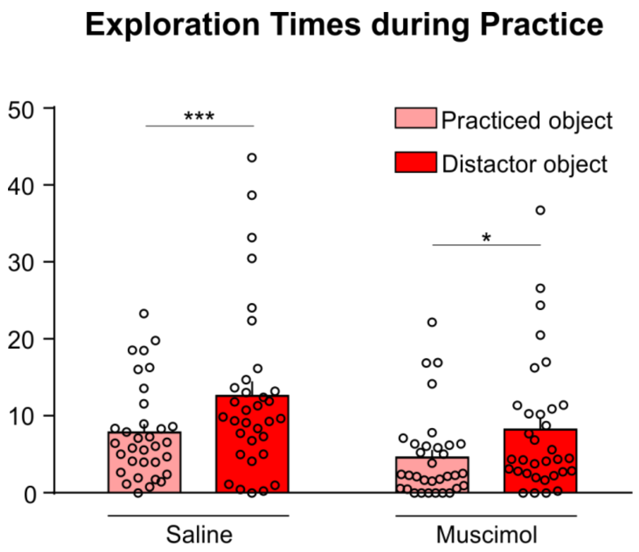

Supplementary Figure 2. (A) For the Saline and Muscimol conditions, average absolute exploration times in seconds during the practice phase for the practiced object A and distractor objects X, Y and Z, across the three practice trials. Values are means ± SEM. ***p=0.0006, t_{32}=3.82, d=0.50 (saline); *p=0.012, t_{32}=2.64, d=0.54 (muscimol); one-tailed paired *t* test, n=33.

Supplementary Methods:

Arena 1 was 50 cm wide x 50 cm long x 39 cm high with black plywood walls and floor, divided into 9 squares by white lines.

Arena 2 was a 60 cm wide x 40 cm long x 50 cm high acrylic box. The floor and two of the walls were white, presenting different visual cues, geometric forms or strips made with self-adhesive paper tape of different colors The frontal wall was transparent and the back wall was hatched.

Arena 3 was 50 cm diameter x 50 cm high round with brown acrylic walls and black plywood floor, divided into 9 squares by white lines.

Arena 4 was a 50 cm wide x 50 cm long x 40 cm high box constructed with white Plexiglas, . Each wall had different visual cues, made with self-adhesive paper tape of different colors.

Arena 5 was 40 cm diameter x 50 cm high round with brown acrylic walls and sky blue floor.

Arena 6 was a bow-tie-shaped maze made of opaque white Plexiglas. It was 94 cm long, 50 cm wide and 50 cm high. Each end of the apparatus was triangular, the apexes of which were joined by a narrow corridor (14 cm wide).

Arena 7 was a Y-shape apparatus constructed from Plexiglas. All walls were 40 cm high, and each arm was 27 cm long and 10 cm wide.

Arena 8 was a triangule of 60 cm wide x 60 cm long x 60 cm high made of white semi-rigid PVC.

Objects

All experiments used numerous junk objects, differing in shape, texture, size, and color. The height of the objects ranged from 8cm to 24 cm. All objects had duplicates so that identical objects could be used at the same time. They were affixed to the floor with an odorless reusable adhesive to prevent them for being displaced. Specific objects were never repeated across different conditions for a given animal. Objectswere cleaned with 50% alcohol wipes after each session except for the ones presented during the shaping phase (see experiment 3 below) in which they were only cleaned between subjects.

Memory Test for Retrieval-Induced Forgetting

<u>The general retrieval practice paradigm:</u>

The retrieval practice paradigm generally involved three conditions: retrieval practice (RP), interference control (IC) and time control (TC). Every condition followed the same basic sequence across three days: Day 1: Habituation to the arenas, Day 2: Habituation to “distractor” objects to be used during the retrieval practice phase of the experiment, and Day 3: The main memory task. During day 3, encoding and testing took place in a single session incorporating the three key phases: encoding, retrieval practice (or an equivalent delay), and final test phases. **Retrieval Practice (RP) condition**: On the first day, each animal was habituated for 10 minutes to two arenas (e.g., arenas 1 and 2) . On day two, each rat was exposed in arena 2 to three pairs of identical distractor objects (X, Y and Z) for 5 min during three consecutive (30 min apart) sessions. The

following day, the main experiment was conducted in arena 1. The **encoding** phase consisted of two sessions separated by 20 minutes in which the animal was allowed to freely explore for 5 min two identical copies of two novel objects: e.g., object A (session 1) and object B (session 2). The **practice** phase took place 30 minutes after the last encoding session and consisted of three 3-min sessions with an inter-session interval (ISI) of 15 min. In each session the animal was exposed to a copy of one of the two objects (A) presented during the encoding phase--accompanied by one copy of the contextually novel objects X, Y or Z (e.g., A & X; then A& Y; then A& Z across the three sessions). We pseudo-randomly assigned which object was practiced from the objects of the encoding phase (either A or B), so the practiced object could either be the first or the second one of the encoding phase. Moreover, the location (right or left) in which the practiced object appeared was randomly assigned for each session. The **test** phase was conducted 30 minutes after the last practice session. The animal was exposed for 3 min to a copy of a non-practiced competitor object presented only during the encoding phase (B) and one completely novel object (C). Fifteen minutes later the animal was re-introduced in the arena and exposed for 3 minutes to a copy of the practiced object (A) and one completely novel object (D). For both test sessions the location of the novel and familiar objects (right or left) were randomly assigned. The letters used in these descriptions and in our diagrams are meant to identify the nature of the item--practiced object, competitor object, novel object or distractor. Repetitions of the same letter across conditions do not indicate that the same object was used across conditions: in fact, different objects were used for the different conditions –RP, IC or TC- of the task.

Interference Control (IC) condition: days 1 and 2 were identical to the RP condition. On the final day, the **encoding** phase was identical to the one in the RP condition. The **practice** phase took place 30 minutes after the encoding phase. and each animal was allowed to explore two copies of objects X, Y and Z during three consecutive 3-min sessions with a ITI of 20 min. The **test** phase was conducted as in the RP condition.

Time Control (TC) condition: days 1 and 2 and the encoding phase were identical to the RP and IC conditions. . There was no practice phase. Instead, the rats spent the same interval of time in their home cages in between the encoding and the test phase. The **test** phase took place at the end of this two hour interval and was conducted as in the two other conditions.

Quantification of behavior: Exploration of each object was defined as the animal directing its nose to the object at a distance of <2 cm and/or touching it with its nose. Turning around or sitting on the object was not considered exploratory behavior. Based on these criteria, we calculated a discrimination index (DI) for each trial of each session on each condition as the difference in time spent exploring the novel and familiar objects divided by the total time spent exploring the objects (i.e. [novel – studied]/total exploration time]). In the case that both objects were identical or both equally novel, we computed the difference between the object on the right and the object on the left. Experimenters were blind to experimental conditions, except for the practice phase. Time spent exploring the object was recorded using manual chronometers. Unlike the live manual recording done in Experiments 1-5, in experiments 6 & 7, each behavioral session was recorded using Samsung HMX-F80 cameras. The cameras were located on top of each arena allowing the visualization of the complete space. Offline analysis was done by a trained person using manual chronometers.

<u>Specific design features of individual experiments</u>: Retrieval-Induced Forgetting Effect (Experiment 1): 14 rats were used in this experiment. Two animals were excluded from the analysis based on a side bias consistent across the entire encoding phase and at least the first trial of the practice phase. The procedure was identical to the standard procedure described above.

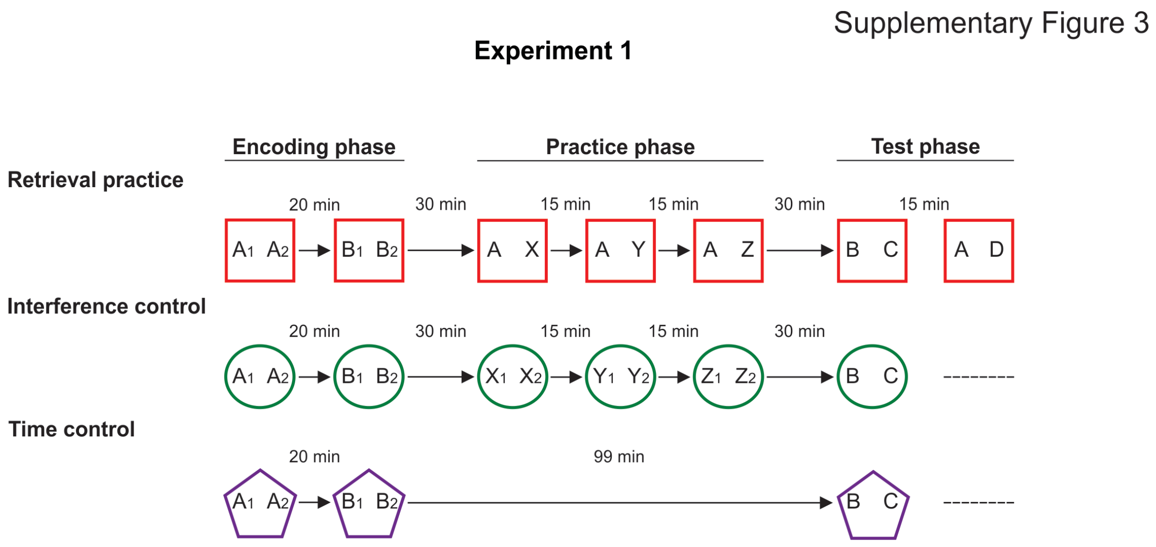

Supplementary Figure 3. Schematic design of Experiment 1 that corresponds to Figure 1.

Retrieval specificity experiment (Experiment 2): 16 rats were assigned for this experiment. Four animals were excluded, three of them showed consistent bias to one of the sides of the arena in encoding phase 1 across all the conditions and the fourth one showed a consistent side bias in all three conditions during encoding phase 2. The design was similar to that of the first experiment, except that a new pair of objects (i.e., two copies of one object known as the "new competitor") was presented to the animals after the practice phase (for the RP and IC conditions) or one hour after the encoding phase (for the TC condition). The test phase took place 30 minutes after the presentation of the new competitor objects (J). For each condition the test phase consisted of a first session in which the memory for the practiced object was evaluated, followed by a test session for the memory of the new competitor (J) (new competitor test).

Supplementary Figure 4

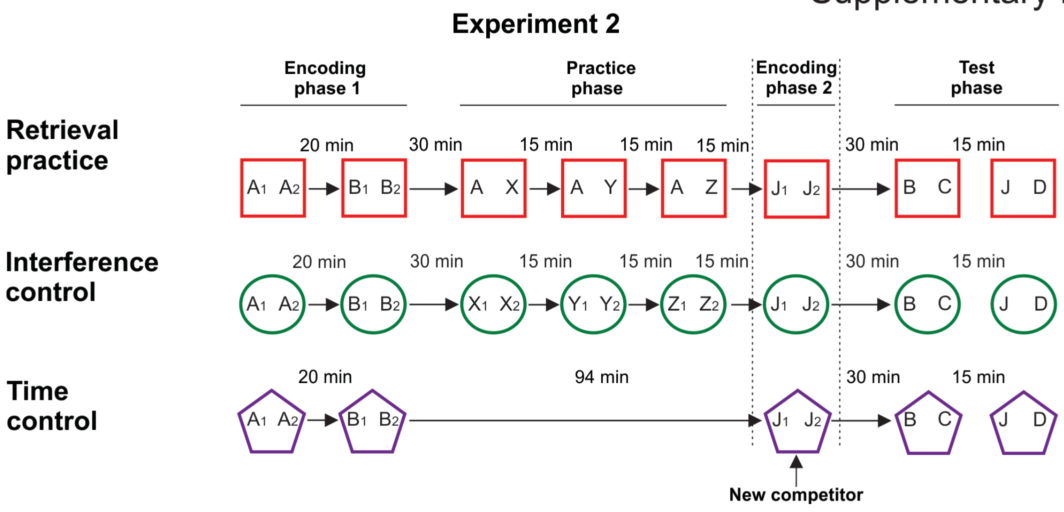

Supplementary Figure 4. Schematic design of Experiment 1 that corresponds to Figure 2.

Cue independence experiment (Experiments 3 and 4): 12 rats were assigned for each of these experiments. One animal was excluded from experiment 3 due to a complete lack of exploration of one of the objects and less than one second of exploration of the other during the test phase 1 of the IC condition. Cue-independence was evaluated as the memory response of the animals to the unpracticed competitor (B) on the final test in the two different arenas in which it had appeared. Because we incorporated a second arena, several additional steps were added.

Shaping: During this pre phase, rats were exposed to two pairs of novel objects in two arenas. The animals were exposed twice to each arena (four sessions lasting 5 min each) with an ISI of 20 minutes during which they encountered the same two pairs of objects in distinct locations. The objects were novel during the first exposure, but familiar during the next three. The location of the objects was always different between the first and the second exposure. This phase was conducted only once during the first week of the experiment independently of the condition. We added this procedure to familiarize rats with the possibility that the very same objects could be presented in different locations within or across arenas. Objects were not cleaned between sessions to encourage rats to recognize that they were the same ones seen before 24 hours later rats were habituated to three different and new arenas (contexts described above). One was used, as in the previous experiments, to familiarize the animals to the “distractor” objects to be presented during retrieval practice. The other two were used for the encoding phase.

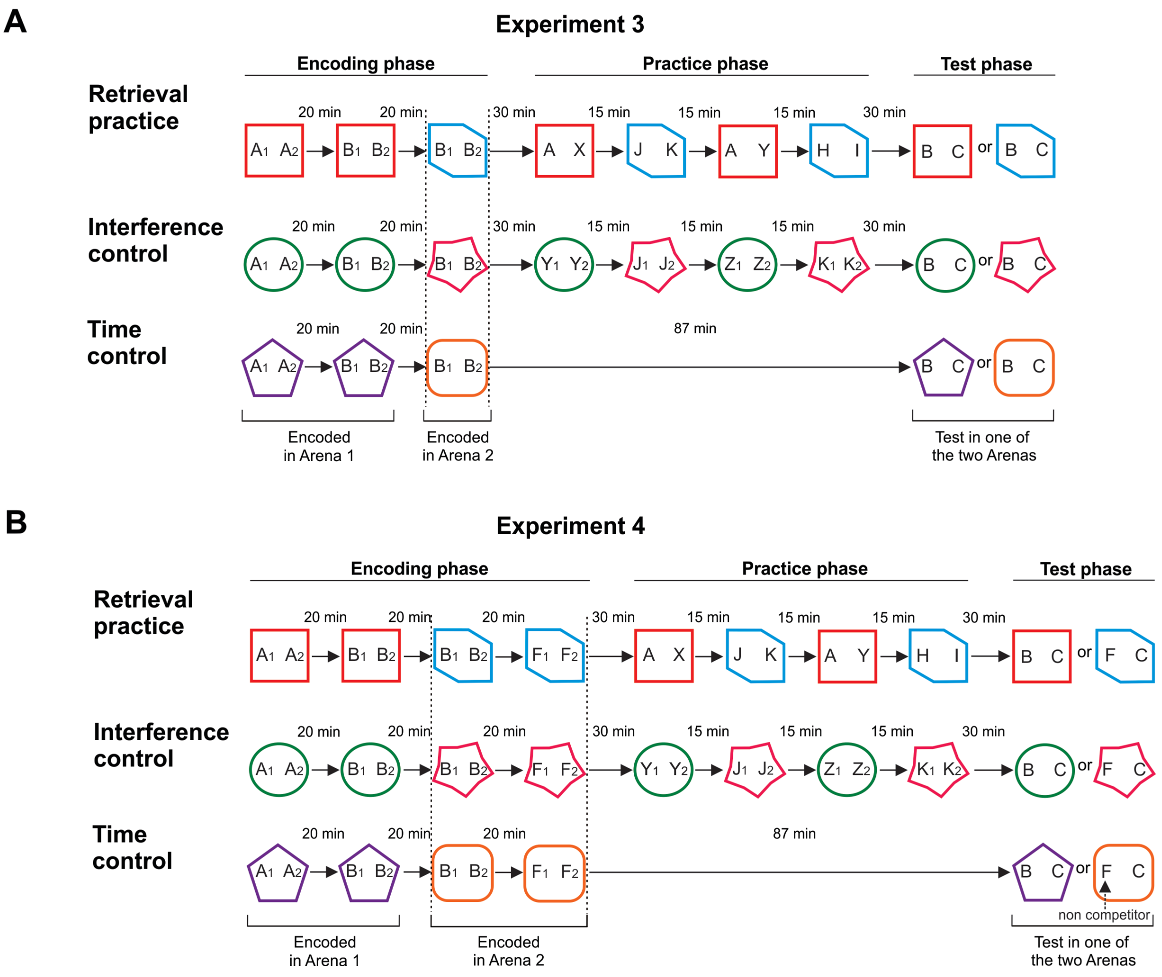

Supplementary Figure 5. (A) Schematic design of Experiment 3 that corresponds to Figure 3C (left panel). (B) Schematic design of Experiment 4 that corresponds to Figure 3C (right panel).

Encoding phase: Each rat had three exposures to two pairs of novel objects for the first experiment (Fig 3A) and four exposures to three pairs of novel objects for the second experiment (Figure 3E). One of the pairs was presented in both arenas (Supplementary Figure 5A). In experiment 4 the third pair was presented in arena 2. **Practice phase**: This phase consisted of two retrieval practice sessions in arena 1, interleaved with two sessions of exposure to two novel objects in arena 2. Each session was 3-min long with an ISI of 20 minutes. We added interpolated presentations of arena 2 to the practice phase in order to equate the frequency and recency with which arenas 1 and 2 were presented in the encoding and practice phases of the experiment. This control should ensure that both arenas are equally accessible and familiar during the test phase.

Test phase: Half the animals were assigned to the “same” arena experimental condition and half to the “different” arena condition. In the "same arena", the final test happened in the practiced

arena (arena 1), whereas in the "different arena", the test took place in the second arena (arena 2). In each case the competitor was presented at test paired with a novel object.

In a second (control) experiment (Experiment 4, Figure 3E, Supplementary Figure 5B), we evaluated rats' memory for an unpracticed object that was uniquely associated with the second unpracticed arena (arena 2). In the test phase, each rat was evaluated for the memory of the two unpracticed objects (B and F), both presented in arena 2 during the encoding phase. One of these unpracticed objects (B) was presented in both arena 1 and arena 2 during the encoding phase and the other (F) was presented only in arena 2.

Durability (Experiment 5): 8 rats were used for this experiment. We replicated the design for Experiment 1 the test phase was separated from the encoding and practice phases by 24 hours (Supplementary Fig 6A).

Involvement of the mPFC (Experiment 6): 13, 12 and 14 rats were used for the RP, IC and TC respectively. Two animals were excluded from the RP group due to a bias toward one side of the arena during both trials of the encoding phase. Two were excluded from the IC group, one because it was mistakenly re-assigned to the saline condition and the second due to a consistent bias towards one side of the arena during all trials of the practice phase. Three animals were excluded from the TC group: (one due to a wrong location of the cannula; the second for diminished exploratory activity during the encoding phases for both conditions, and the third due to blockade of one of the cannulae during the second trial).

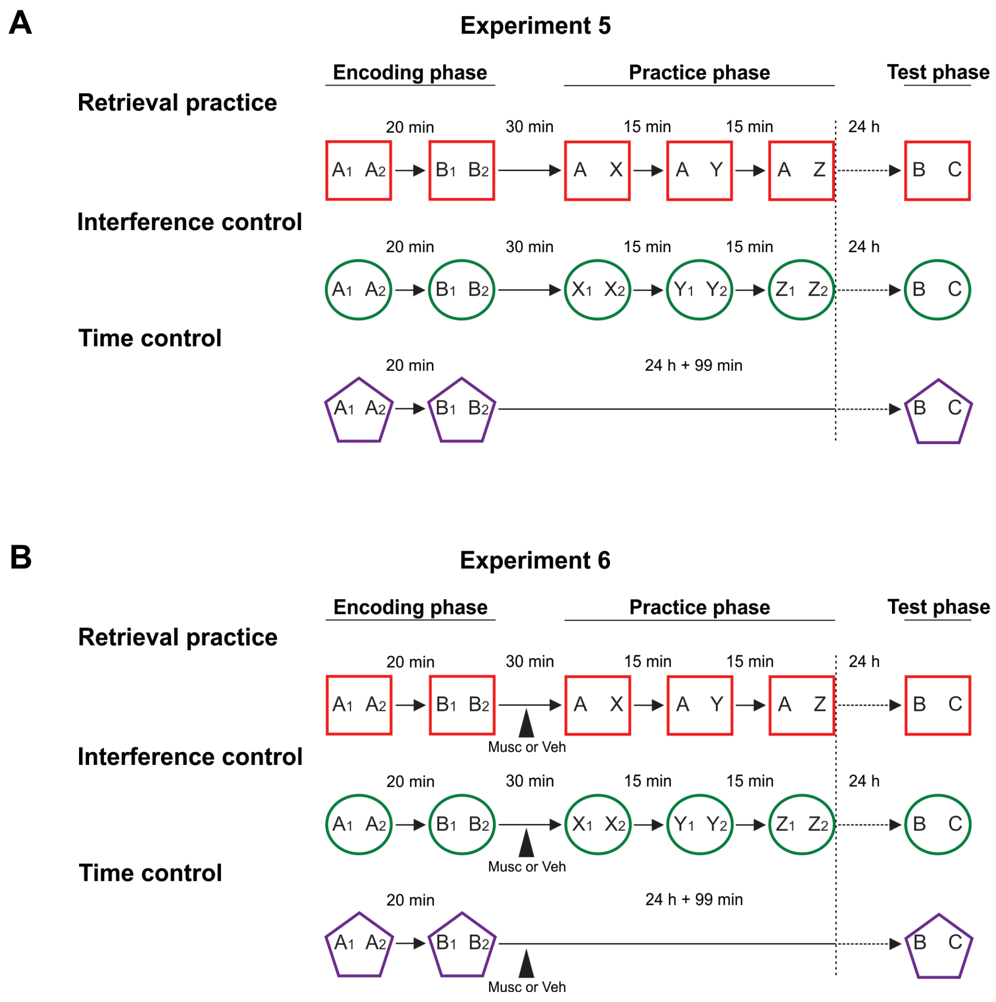

Supplementary Figure 6. (A) Schematic design of Experiment 5 that corresponds to Figure 4A. (B) Schematic design of Experiment 6 that corresponds to Figure 4D.

Surgery: Rats were deeply anesthetized with ketamine (60 mg/kg) and xylazine (8 mg/kg) and put in a stereotaxic frame. The skull was exposed and adjusted to place bregma and lambda on the same horizontal plane. After small burr holes were drilled, a set of 22 g guide cannulae were implanted bilaterally into the mPFC (AP +3.20 mm/ LL ± 0.75 mm / DV -3.50 mm) (Fig. 5B). andfixed to the skull with dental acrylic. A dummy cannula was inserted to each cannula to prevent clogging. At the end of surgery, animals were injected with a single dose of meloxicam (0.2 mg/kg) as an analgesic.

We used a mixed design: Rats were assigned to one condition (either RP, IC or TC) and were trained and tested twice, once with muscimol and once with vehicle. The order in which animals were infused was randomly assigned. The two sessions were separated by 5 days to leave time for the drugs to washout.

Behavioral procedures commenced 5-7 days after surgery. On the experimental day, the dummy cannulas were removed before the injection and an injection cannula extending 1mm below the guide cannula was inserted. The injection cannula was connected to a 10 µl Hamilton syringe and rats received bilateral 1 µl infusions of muscimol (0.1 µg/µl) or vehicle into the mPFC 15 minutes before the retrieval practice phase (or at the corresponding points in the IC and TC conditions). We conducted the final test 24hs later (Supplementary Figure 6B).

Immunohistochemistry (Experiment 7): 7 rats were used for each condition. Because c-Fos expression can only be tested once per animal, rats could only participate in one of the three main conditions (RP, TC, or IC conditions). Animals were randomly assigned to one of five conditions. A key goal was to ensure that c-Fos expression was mainly associated with the practice task. Thus, we separated the encoding and practice phases by 24 hr to avoid c-Fos expression contamination from the encoding into the practice phase. To evaluate the role of the mPFC at different stages of the practice phase, we separated the three sessions of practice in a way that allowed us to measure the contribution of the first two sessions on c-Fos expression in the mPFC compared to the contribution of the third one. 90 minutes after either the second or the third practice session, animals were tested and immediately anesthetized and perfused transcardialy with saline followed by paraformaldehyde 4 %. Brains were preserved in 30% sucrose and then cut using a freezing microtome into 35 µm sections (See schematic design in Supplementary Fig. 7).

Supplementary Figure 7

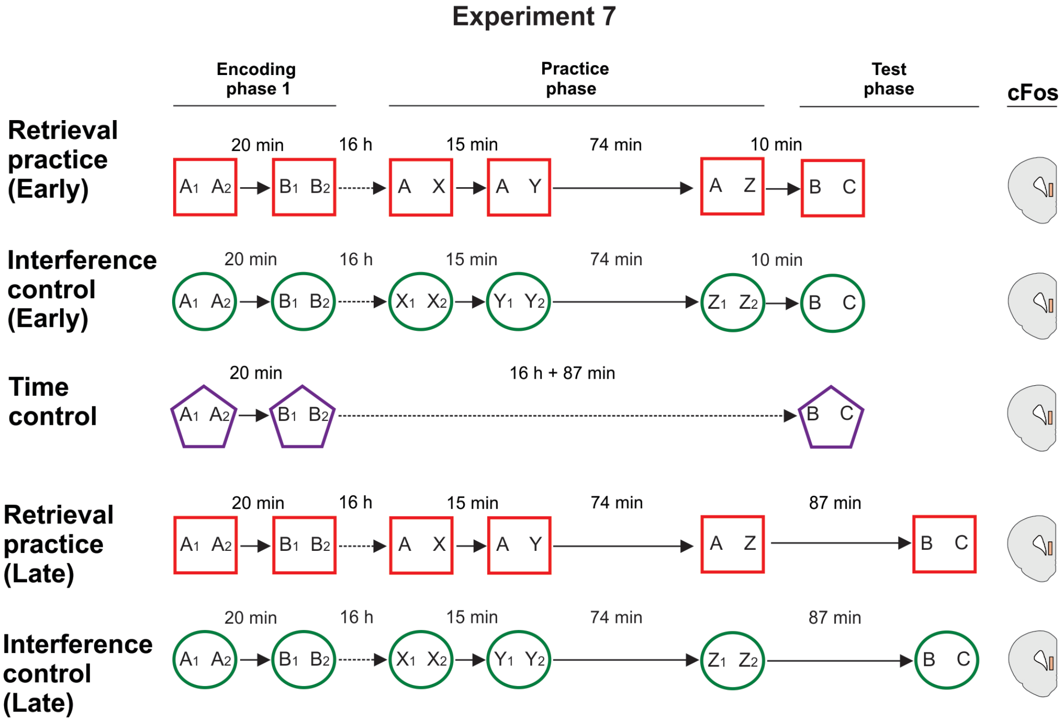

Supplementary Figure 7. Schematic design of Experiment 7 that corresponds to Figure 7.

Peroxidase-immunohistochemical staining was performed on free-floating sections. Sections were washed twice in phosphate-buffered saline (PBS 0.1 M, pH 7.4) and treated with 0.6% H_2O_2 in PBS for 30 min, washed four times in PBS followed by incubation in blocking solution (2% normal goat serum in PBS with 0.4% Triton X-100) for 1 h. Sections were incubated for 18 hrs with a polyclonal primary antibody (c-Fos antibody, SC-52, Santa Cruz Biotechnology diluted 1:3500), containing 2% goat normal serum (Jackson ImmunoResearch), diluted in PBS/Triton. Sections were then incubated with the biotinylated secondary antibody (1:1000; Jackson), also diluted in PBS/Triton for 2 hours at room temperature. Sections were washed and incubated in avidin-biotin-peroxidase solution (ABC Elitekit, Vector Labs, Burlingame, CA, USA) for 60 min. The reaction was developed by the addition of 2.5% diaminobenzidine tetrahydrochloride (Sigma, St. Louis, MO, USA) and 0.08% H_2O_2 in 0.1M phosphate buffer, pH7.4. Sections were washed (three times, 5min) with 0.1M phosphate buffer(pH7.4). Afterwards, the sections were mounted, dried, dehydrated in a graded alcohol series, cleared in xylene, and coverslipped with Canada mounting solution.

Quantification: Positive nuclei quantitative analysis was performed as described by Sacco and Sacchetti (2010)[1]. Medial prefrontal cortex was anatomically defined according to the atlas of Paxinos & Watson[2]. Images were obtained using an Olympus BX53 microscope (Zeiss; X10 objective) equipped with a digital camera interfaced with QCapture imaging software. c-Fos-positive nuclei were analyzed bilaterally using serial sections of mPFC (AP from = +3.20 mm to +2.20). c-Fos-positive nuclei were counted by an experimenter blind to experimental conditions using Image J software (http://rsb.info.nih.gov/ij/).

Supplementary Tables

Supplementary Table 1: Retrieval practice exploration times for experiment 1

	**Retrieval Practice**				**Interference Control**				
	A	X/Y/Z	DI	p_{DI}	$X_1/Y_1/Z_1$	$X_2/Y_2/Z_2$	DI	p_{DI}	N
**Session 1**	7.21 ± 1.41	11.29 ± 2.86	0.24 ± 0.10	0.037	11.43 ± 2.67	12.05 ± 2.75	0.06 ± 0.02	0.72	12
**Session 2**	6.21 ± 1.57	11.17 ± 2.26	0.33 ± 0.13	0.031	9.65 ± 2.18	12.03 ± 2,99	0.14 ± 0.12	0.25	12
**Session 3**	3.25 ± 0.67	13.28 ± 2.67	0.44 ± 0.12	0.003	8.98 ± 2.46	10.33 ± 1.86	0.11 ± 0.10	0.32	12

Supplementary Table 1: Absolute exploration time during the retrieval practice phase for experiment 1. Total exploration times in seconds during the practice phase for the RP and IC conditions. Values are expressed in seconds (mean ± S.E). The discrimination index (DI) was calculated as the time spent exploring the distractor object (X, Y or Z) minus the time spent exploring the practiced object A over the total exploration time during the session (A+distractor) for the RP condition. For the IC condition, the DI was calculated as the time exploring one of the objects (e.g., X_1) minus the time exploring the other object (e.g., X_2) of the sum of the two exploration times (X_1+X_2). DIs were significantly different from zero in each of the three retrieval practice sessions for the RP condition, but not in the IC condition (one-sample t test against a theoretical value of 0. Significance levels are indicated as "p_{DI}"). Contrasts were considered significant if p <0.05.

Supplementary Table 2: Encoding exploration times for experiment 2

	**A_1**	**A_2**	**p**	**B_1**	**B_2**	**p**	**J_1**	**J_2**	**p**	**N**
**RP**	31.06 ± 4.00	31.09 ± 4.67	0.99	25.20 ± 3.13	23.69 ± 3.72	0.57	12.07 ± 3.40	14.14 ± 2.79	0.50	12
**IC**	25.75 ± 3.56	24.53 ± 3.47	0.29	20.98 ± 3.18	25.27 ± 3.66	0.20	16.99 ± 4.40	13.27 ± 2.68	0.20	12
**TC**	26.20 ± 4.45	27.21 ± 3.83	0.43	27.46 ± 4.43	29.66 ± 4.82	0.44	19.26 ±2.79	19.89 ± 3.04	0.80	12

Supplementary Table 2: Absolute exploration time during the encoding phase for experiment 2. Total exploration times during encoding phase 1 (objects A and B) and encoding phase 2 (object J) for the retrieval practice (RP), interference control (IC) and time control (TC) conditions. A, B and J represent the different objects used as indicated in Fig 2A. Values are expressed in seconds (mean ± S.E). Contrasts were considered significant if p < 0.05, Student´s test for each encoding phase (e.g., A_1 vs. A_2) and for the total exploration time (e.g A_1+A_2 vs. B_1+B_2 vs. J_1+J_2). We found a significant reduction in total exploration of the new competitor J during encoding 2 compared with exploration of A and B during encoding 1 for the RP and BS conditions (repeated measures one-way ANOVA followed by post-hoc Bonferroni contrasts, p<0.01 for A (total) vs. J (total) and p<0.05 for B (total) vs. J (total)). For the TC condition J (total) was significantly different from B (total), but not from A (total), p<0.05).

Supplementary Table 3: Exploration times during final test for experiment 2

	Object B	Object C	p	Object J	Object D	p	N
**RP**	14.94 ± 3.49	11.51 ± 1.69	0.305	4.25 ± 1.10	9.92 ± 2.41	0.025	12
**IC**	6.259 ± 1.44	16.11 ± 2.92	0.002	11.15 ± 3.06	25.56 ± 5.09	0.024	12
**TC**	13.25 ± 2.53	28.19 ± 4.17	0.001	6.09 ± 1.62	16.05 ± 5.11	0.068	12

Supplementary Table 3: Absolute exploration time during the final test for experiment 2. Total exploration scores during the test phase for the RP, IC and TC conditions. Values are expressed in seconds (mean ± S.E). Each animal was exposed to two different final tests. The first test compared the exploration time of the competitor object B against a novel object C, while the second test compared the new competitor object J against a novel object D. We found that exploration of the new competitor object J was significantly lower than exploration of a novel object D for the three conditions, while exploration of competitor object B was not different from exploration of a novel object C for the RP condition. Contrasts were considered significant if $p < 0.05$, Student´s t test.

Supplementary Table 4: Encoding exploration times for experiment 3

	A_1	A_2	p	B_1	B_2	p	B_1'	B_2'	p	N
**RP**	23.36 ± 2.05	20.67 ± 2.13	0.07	21.01 ± 2.24	20.82 ± 2.16	0.81	17.39 ± 1.69	18.28 ± 1.64	0.21	23
**IC**	21.48 ± 2.92	21.54 ± 2.95	0.96	18.11 ± 2.30	17.92 ± 2.22	0.80	16.05 ± 1.63	16.18 ± 2.06	0.87	23
**TC**	19.93 ± 2.01	21.02 ± 1.63	0.26	19.38 ± 2.19	20.33 ± 2.26	0.41	17.72 ± 1.51	17.21 ± 1.63	0.63	23

Supplementary Table 4: Absolute exploration times during the encoding phase for experiment 3. Total exploration times during the encoding phase for the RP, IC and TC conditions for the animals that were finally tested in **arena 1** (practiced arena) or in **arena 2** (unpracticed arena). B and B' represent the same objects. B represents the values when they were presented in arena 1 and B' when presented in arena 2. Values are expressed in seconds (mean ± S.E). Contrasts were considered significant if $p < 0.05$, Student´s t test. Addition of a third session to the encoding phase did not reduce exploration of the encoded objects.

Supplementary Table 5: Exploration times during the practice phase for experiment 3

	Retrieval Practice				Interference Control				
	A/J/ A/H	X/K/Y/I	DI	p_{DI}	$X_1/J_1/Y_1/K_1$	$X_2/J_2/Y_2/K_2$	DI	p_{DI}	N
**Session 1 Arena 1**	12.02 ± 1.77	18.37 ± 1.95	0.21 ± 0.05	0.0007	11.49 ± 1.40	11.77± 1.40	-0.02 ± 0.04	0.56	23
**Session 2 Arena 2**	18.32 ± 2.29	16.67 ± 1.56	-0.03 ± 0.04	0.3600	15.90 ± 1.76	16.65 ± 2.34	-0.03± 0.04	0.51	23
**Session 3 Arena 1**	8.39 ± 1.11	14.99 ± 1.78	0.25 ± 0.05	0.0002	9.87 ± 1.42	11.24± 1.70	0.10 ± 0.05	0.04	23
**Session 4 Arena 2**	17.95 ± 2.03	15.65 ± 2.04	-0.13 ± 0.07	0.0600	15.80 ± 1.90	15.58 ± 2.50	-0.10 ± 0.07	0.16	23

Supplementary Table 5: Absolute exploration times during the practice phase for experiment 3. Total exploration times during the retrieval practice phase for the RP, and IC condition of the animals that were finally tested in arena 1 or arena 2. Values are expressed in seconds (mean ± S.E). Discrimination index (DI) was calculated as the time spent exploring the distractor object minus the time spent exploring the practiced object A over the total exploration time during the session (A+distractor) for the RP condition. For the IC condition, the DI was calculated as the time exploring one of the objects (e.g., X_1) minus the time exploring the other object (e.g., X_2) of the sum of the two exploration times (X_1+X_2). A one-sample t test was calculated for the DI against a theoretical value of 0. Contrasts were considered significant if $p < 0.05$. Interleaving trials during the practice phase did not affect recognition of the practiced object A during the two practice trials in which it was presented.

Supplementary Table 6: Exploration times during the test phase for experiment 3

	Arena 1				Arena 2			
	**Object B**	**Object C**	**p**	**N**	**Object B**	**Object C**	**p**	**N**
**RP**	14.97 ± 1.89	17.16 ± 3.36	0.410	11	11.93 ± 2.44	13.09 ± 2.55	0.260	12
**IC**	6.40 ± 1.80	15.73 ± 3.95	0.004	11	5.05 ± 0.96	13.07 ± 2.58	0.001	12
**TC**	10.70 ± 2.05	21.52 ± 3.68	0.008	11	5.54 ± 0.77	15.87 ± 3.19	0.002	12

Supplementary Table 6: Absolute exploration times during the final test phase for experiment 3. Exploration times during the final test were not affected by the change in the encoding and the practice phases of the protocol. Total exploration times during the final test phase for the RP, IC and TC conditions. Values are expressed in seconds (mean ± S.E). Contrasts were considered significant if $p < 0.05$, Student´s t test.

Supplementary Table 7: Encoding exploration times for experiment 4

	A_1	A_2	p	B_1	B_2	p	B_1'	B_2'	p	C_1	C_2	p	N
**RP**	31.95 ± 3.63	27.62 ± 4.45	0.39	27.07 ± 4.64	25.55 ±3.76	0.52	21.27 ±3.76	20.38 ± 3.37	0.52	23.11 ± 3.64	26.19 ± 3.54	0.30	8
**IC**	26.08 ± 5.76	19.44 ± 2.14	0.19	21.84 ± 4.00	23.26 ± 7.11	0.70	19.35 ± 3.44	23.18 ± 4.49	0.09	20.84 ± 3.33	17.86 ± 2.91	0.29	8
**TC**	22.34 ± 2.60	19.23 ± 2.71	0.06	26.74 ± 5.26	20.41 ±5.37	0.05	21.49 ± 1.36	20.76 ± 1.12	0.68	22.09 ± 3.92	15.89 ± 3.51	0.03	8

Supplementary Table 7: Absolute exploration time during the encoding phase for experiment 4. Addition of two trials during encoding did not affect exploration of the newly encoded objects. Total exploration times in seconds during encoding phase for the RP, IC and TC. B and B' represent the same objects. F represents a novel object that was only presented in arena 2. Values are expressed in seconds (mean ± S.E). Contrasts were considered significant if $p < 0.05$, Student's t test.

Supplementary Table 8: Exploration times during the final test phase for experiment 4

	Competitor Object				Non-Competitor Object			
	**B**	**C**	**p**	**N**	**F**	**G**	**p**	**N**
**RP**	11.91 ± 2.05	13.62 ± 2.26	0.2000	8	7.67 ± 0.87	13.78 ± 1.07	0.0004	8
**IC**	7.48± 2.18	16.25± 3.03	0.0002	8	8.06 ± 2.10	15.26 ± 4.38	0.0570	8
**TC**	5.97 ± 1.51	17.70 ± 4.42	0.0100	8	8.43 ± 1.74	19.72 ± 6.32	0.0700	8

Supplementary Table 8: Absolute exploration time during the final test phase for experiment 4. Total exploration times during test phase for the RP, IC and TC condition. Values are expressed in seconds (mean ± S.E). On the left the competitor object B was presented in arena 1 together with a novel object C. On the right the non-competitor object F was presented in arena 2 together with a complete novel object G. Contrasts were considered significant if $p < 0.05$, Student´s t test. Encoding a third novel object during the encoding phase did not change the animals' novelty preference during the final test.

Supplementary Table 9: Exploration times during the final test phase for experiment 5

	Object B	Object C	p	N
**RP**	19.96 ± 2.58	19.93 ± 2.06	0.9800	8
**IC**	8.78 ± 1.60	18.92 ± 3.12	0.0006	8
**TC**	12.98 ± 2.64	26.74 ± 4.08	0.0012	8

Supplementary Table 9: Absolute exploration time during the test phase for experiment 5. Total exploration times during test phase for the RP, IC and TC conditions. Values are expressed in seconds (mean ± S.E). Contrasts were considered significant if $p < 0.05$, Student´s t test. Delaying the final test by 24 hr did not affect novelty preference.

Supplementary Table 10: Exploration times during the practice phase in the Retrieval Practice condition for experiment 6

	Saline		Muscimol			
	A	X/Y/Z	A	X/Y/Z	p_{total}	N
Session 1	10.79 ± 2.06	14.03 ± 2.03	6.32 ± 1.23	10.26 ± 2.46	0.21	11
Session 2	7.24 ± 1.70	13.67 ± 4.01	5.59 ± 2.48	7.78 ± 2.35	045	11
Session 3	5.55 ± 1.51	10.15 ± 3.52	2.86 ± 1.51	7.29 ± 3.12	0.33	11

Supplementary Table 10: Absolute exploration times during the retrieval practice phase for experiment 6 (retrieval practice condition). Total exploration times during the retrieval practice phase for the RP group when animals were infused with saline (left) or muscimol (right). Values are expressed in seconds (mean ± S.E). Contrasts were considered significant if $p < 0.05$, Student's t test, comparing total exploration time between saline- and muscimol-injected animals for each retrieval practice session (e.g A+X muscimol vs. A+X saline). Significance level is indicated as "p_{total}". Muscimol injection did not affect total exploration times during the practice phase compared to saline injection.

Supplementary Table 11: Exploration times during the practice phase in the Interference Control condition for experiment 6

	Saline		Muscimol			
	$X_1/Y_1/Z_1$	$X_2/Y_2/Z_2$	$X_1/Y_1/Z_1$	$X_2/Y_2/Z_2$	p_{total}	N
Session 1	10.82 ± 4.30	11.23 ± 3.36	6.47 ± 1.96	7.17 ± 2.05	0.33	10
Session 2	9.45 ± 1.92	9.50 ± 1.89	7.46 ± 1.90	6.51 ± 1.40	0.32	10
Session 3	4.38 ± 1.72	5.40 ± 1.62	3.73 ± 1.44	5.52 ± 1.61	0.91	10

Supplementary Table 11: Absolute exploration times during the retrieval practice phase for experiment 6 (Interference control condition). Total exploration times during the practice phase for the IC group when animals were infused with saline (left) or muscimol (right). Values are expressed in seconds (mean ± S.E). Contrasts were considered significant if $p < 0.05$, Student's t test, comparing total exploration time between saline- and muscimol-injected animals for each practice session (e.g X_1+X_2 muscimol vs. X_1+X_2 saline).). Significance level is indicated as "p_{total}". Muscimol injection did not affect total exploration times during the practice phase compared to saline injection.

Supplementary Table 12: Exploration times during the final test phase for experiment 6

	Saline			Muscimol			
	**Object B**	**Object C**	**p**	**Object B**	**Object C**	**p**	**N**
**RP**	14.07 ± 2.74	14.02 ± 2.22	0.980	7.16 ± 2.40	17.80 ± 4.65	0.0070	11
**IC**	10.56 ± 1.82	22.13 ± 4.31	0.009	7.74 ± 1.73	20.77 ± 4.00	0.0006	10
**TC**	10.43 ± 1.84	19.11 ± 2.87	0.001	8.53 ± 1.78	20.81 ± 4.50	0.0026	11

Supplementary Table 12: Absolute exploration time during the final test phase experiment 6. Total exploration times during the final test phase for the RP, IC and TC conditions. Values are expressed in seconds (mean ± S.E). Contrasts were considered significant if $p < 0.05$, Student´s t test. Muscimol injection before the practice phase did not affect exploration times during the final test phase compared to saline injection.

Supplementary Table 13: Exploration times during the practice phase for experiment 7

	RP (Early)			RP (Late)			IC (Early)			IC (Late)		
	**A**	**X/Y/Z**	**p**	**A**	**X/Y/Z**	**p**	**$X_1/Y_1/Z_1$**	**$X_2/Y_2/Z_2$**	**p**	**$X_1/Y_1/Z_1$**	**$X_2/Y_2/Z_2$**	**p**
**Session 1**	11.77± 2.22	23.12± 5.14	0.014	10.37± 2.3	17.30± 3.76	0.006	12.26 ± 2.52	15.18 ± 3.52	0.18	13.41 ± 4.60	14.93± 5.08	0.54
**Session 2**	9.33 ± 1.46	18.67± 3.30	0.010	11.42± 2.30	18.85± 5.0	0.090	13.91 ± 2.46	13.36± 2.80	0.59	18.84 ± 2.76	16.65± 2.36	0.37
**Session 3**	8.96 ± 1.48	15.55± 2.28	0.001	10.90± 3.68	16.20± 4.26	0.040	7.24± 1.26	8.57± 1.53	0.31	12.07 ± 2.69	8.28 ± 2.00	0.01

Supplementary Table 13: Absolute exploration time during the practice phase for experiment 7. Total exploration times during the retrieval practice phase for the Early and Late treatments in every condition (RP, IC and TC). Values are expressed in seconds (mean ± S.E). Delaying the retrieval practice phase by 24 hr and separating the third retrieval practice session did not affect recognition of the practiced object A. Contrasts were considered significant if $p < 0.05$, Student´s t test.

Supplementary Table 14: Exploration times during the final test for experiment 7

	Object B	Object C	p	N
**RP (Early)**	20.99 ± 3.16	20.71 ± 2.52	0.95	7
**RP (Late)**	16.90 ± 3.88	18.21 ± 6.09	0.68	7
**IC (Early)**	10.25 ± 2.95	22.10 ± 5.17	0.01	6
**IC (Late)**	11.88 ± 7.95	26.10 ± 7.95	0.06	7
**TC**	9.02 ± 2.18	18.72 ±6.06	0.07	6

Supplementary Table 14: Absolute exploration time during the final test phase for experiment 7. Total exploration scores during final test phase for the Early and Late treatments in every condition (RP, IC and TC). Delaying the retrieval practice phase by 24 hr and separating the third retrieval practice session did not affect performance during the final test compared with all the other experiments. Values are expressed in seconds (mean ± S.E). Contrasts were considered significant if $p < 0.05$, Student´s t test.

Supplementary references

1. Sacco, T. & Sacchetti, B. Role of secondary sensory cortices in emotional memory storage and retrieval in rats. *Science* **329**, 649-656 (2010).
2. Paxinos, G. & Watson, C. *The rat brain in stereotaxic coordinates* (Academic Press, San Diego, 1998).
